# Fenticonazole nitrate loaded trans-novasomes for effective management of tinea corporis: design characterization, *in silico* study, and exploratory clinical appraisal

**DOI:** 10.1080/10717544.2022.2057619

**Published:** 2022-04-04

**Authors:** Rofida Albash, Maha H. Ragaie, Mahmoud A. El Hassab, Radwan El-Haggar, Wagdy M. Eldehna, Sara T. Al-Rashood, Shaimaa Mosallam

**Affiliations:** a Department of Pharmaceutics, College of Pharmaceutical Sciences and Drug Manufacturing, Misr University for Science and Technology, Giza, Egypt; b Department of Dermatology, STDʼs and Andrology, Faculty of Medicine, Minia University, Al-Minya, Egypt; c Department of Medicinal Chemistry, Faculty of Pharmacy, King Salman International University (KSIU), South Sinai, Egypt; d Pharmaceutical Chemistry Department, Faculty of Pharmacy, Helwan University, Cairo, Egypt; e Department of Pharmaceutical Chemistry, Faculty of Pharmacy, Kafrelsheikh University, Kafr el-Sheikh, Egypt; f Department of Pharmaceutical Chemistry, College of Pharmacy, King Saud University, Riyadh, Saudi Arabia; g Department of Pharmaceutics and Industrial Pharmacy, Faculty of Pharmacy, October 6 University, Giza, Egypt

**Keywords:** Fenticonazole nitrate, Brij^®^, novasomes, *in silico* study, XTT reduction assay, clinical appraisal

## Abstract

The current investigation aimed for loading fenticonazole nitrate (FTN), an antifungal agent with low aqueous solubility, into trans-novasomes (TNs) for management of tinea corporis topically. TNs contain Brij^®^ as an edge activator besides the components of novasomes (cholesterol, Span 60, and oleic acid) owing to augment the topical delivery of FTN. TNs were fabricated applying ethanol injection method based on D-optimal experiment. TNs were evaluated with regard to entrapment efficiency percent (EE%), particle size (PS), polydispersity index (PDI), and zeta potential (ZP). Further explorations were conducted on the optimum formulation (F7). F7 showed spherical appearance with EE%, PS, PDI, and ZP of 100.00 ± 1.10%, 358.60 ± 10.76 nm, 0.51 ± 0.004, and −30.00 ± 0.80 mV, respectively. The *in silico* study revealed the ability of the FTN–cholesterol complex to maintain favorable interactions throughout the molecular dynamics simulation (MDS) study. Moreover, *Trichophyton mentagrophytes* growth was inhibited effectively by F7 than by FTN suspension applying 2,3-bis(2-methyloxy-4-nitro-5-sulfophenyl)-2H-tetrazolium-5-carboxanilide (XTT) reduction assay. Furthermore, a clinical appraisal on patients with tinea corporis fungal lesions confirmed the superiority of F7 compared to Miconaz^®^ cream in the magnitude of clinical cure of tinea corporis. Thereby, TNs could be considered as promising vesicles for enhancing the antifungal potential of FTN for the topical management of tinea corporis.

## Introduction

Fungal diseases are becoming more common these days. They have greater toxic side effects encountered with traditional systemic therapy (Kumar et al., 2014). Tinea corporis, also known as ringworm, is a dermatophytosis (superficial fungal infection especially on the skin) (Merad et al., 2021). The therapeutic efficacy of medication applied topically is mainly determined by its capability to enter and penetrate the skin. Thereby, the development of an innovative drug delivery system will produce better outcomes owing to passing the stratum corneum (SC) and targeting the site of infection (Mosallam et al., 2021a). Fenticonazole nitrate (FTN) is an antifungal agent that belongs to imidazoles. It works by blocking ergosterol production and therefore damaging the cell membrane (Campos et al., 2018). FTN has both fungistatic and fungicidal properties against yeasts, fungi, and dermatophytes. It also inhibits the growth of gram-positive bacteria (Jung et al., 1988). Hence, FTN is thought to be a promising topical agent for treating skin fungal infections. Unfortunately, the low aqueous solubility of FTN (<0.10 mg/mL) (Albash et al., 2020) arouses the need for designing a new vesicular system to deliver FTN effectively and compel cure of fungal infections.

Novasome technology is a patented and innovative encapsulation technique created by IGI Laboratories NOVAVAX to address efficacy issues with conventional drug delivery methods. Novasomes are thought to have improved niosomal or liposomal structure. They are generally composed of fatty acid, cholesterol, and monoester of polyoxyethylene fatty acid (Singh et al., 2011; Mosallam et al., 2021a). Several novasomes-based vaccines have been authorized (Gregoriadis, 1995; Chambers et al., 2004). In the current work, to augment the potential of novasomes in the topical delivery of FTN for treatment of tinea, Brij^®^ was incorporated as an additional penetration enhancer in the novasomes structure to prepare trans-novasomes (TNs). Brij^®^ is composed of polyethylene gylated single-chain penetration enhancer with different acyl chain entities and polyethylene glycol (PEG) chain lengths (Tagami et al., 2011). PEG moieties might augment the water uptake in the SC resulting in SC swelling as well as SC lipids and/or corneocytes undergo structural changes resulting in the widening of intercellular junctions in the barrier. As a result, the penetration of SC is improved allowing suitably deformed TNs to enter deeply into the epidermal layers (Rangsimawong et al., 2014). In addition, Brij^®^ may affect the interfacial characteristics of novasomes influencing their deposition and extending their residence duration at the site of action (Vega et al., 2013). Moreover, Mosallam et al. (2021a) previously reported that the presence of free fatty acid such as oleic acid had an antifungal effect.

As far as we can tell, this is the first research to employ TNs as a drug carrier to optimize the antifungal potency of FTN, as well as the first to test FTN loaded TNs clinically on patients suffering from tinea corporis. Hence, the goal of this work was to estimate the potential of TNs to optimize the topical delivery of FTN. To accomplish the preceding target, different factors impacting vesicles properties were attempted by D-optimal design applying the Design Expert^®^ program. Span 60 amount (*X*_1_), oleic acid amount (*X*_2_), and Brij^®^ type (*X*_3_) were chosen as independent factors, whereas entrapment efficiency percent (EE%), particle size (PS), polydispersity index (PDI), and zeta potential (ZP) were chosen as dependent responses. The optimum TNs was investigated *in vitro* for its antifungal activity in comparison to FTN suspension utilizing 2,3-bis-(2-methyloxy-4-nitro-5-sulfophenyl)-2H-tetrazolium-5-carboxanilide (XTT) reduction technique. To investigate the antifungal ability of the optimum TNs compared to Miconaz^®^ cream through evaluating their efficiency on patients suffering from tinea corporis.

## Materials

Fenticonazole nitrate was kindly provided by Andalous Pharmaceutical Co. (Cairo, Egypt). Span 60, Brij 93, Brij 58, and cholesterol were purchased from Sigma-Aldrich Chemical Co. (St. Louis, MO). Oleic acid, methyl alcohol, and dimethyl sulfoxide (DMSO) were gained via El-Nasr Chemicals Co. (Cairo, Egypt). Miconaz^®^ cream (miconazole nitrate 2%) was purchased from Medical Union Pharmaceutical (MUP) Co. (Cairo, Egypt).

## Methods

### Formation of FTN-TNs

TNs were fabricated by varying Span 60 amount, oleic acid amount, and Brij^®^ type employing ethanol injection technique (Kakkar & Kaur, 2011). FTN (25 mg), Span 60, oleic acid, and cholesterol (30 mg) were first dissolved in ethanol in a 60 °C water-bath (Type USR3, Julabo Labortechnik, Seelbach, West Germany). Afterward, the ethanolic solution was injected drop wise into a five-folds greater vehicle of distilled water that was magnetically stirred at 60 °C on a magnetic stirrer (model MSH-20D, GmbH, Germany). Previously, the Brij^®^ (10 mg) was mixed with distilled water. When sudden turbidity was detected, TNs were produced. The mixture was constantly mixed until all of the ethanol had evaporated for 30 min. The outcome was subsequently subjected to sonication applying a sonicator water bath using a bath-sonicator (Ultra Sonicator, model LC 60/H, Elma, Singen, Germany) for 5 min for reducing the PS. After that, TNs dispersions were maintained at 4 °C (Al-Mahallawi et al., 2014).

### Characterization of FTN-TNs

#### Assessment of EE%

A cooling centrifuge (Sigma 3-30 KS, Osterode, Germany) was used to centrifuge 1 mL of each TNs dispersion for 1 h at 4 °C and 20,000 rpm. Following that, methyl alcohol was used to destroy the sediment and FTN concentration was determined at *λ*_max_ 252 nm (Albash et al., 2021) via UV/VIS spectrophotometer (Shimadzu UV1650, Spectrophotometer, Kyoto, Japan). FTN EE% was calculated by applying the following equation (Albash et al., 2020):

(1) $$\mathrm{EE}\;(\%)=\frac{\text{entrapped FTN}}{\text{total amount of FTN}}\;\times 100$$

#### Assessment of PS, PDI, and ZP

PS, PDI, and ZP were measured for the fabricated TNs by Zetasizer 2000 (Malvern Instrument Ltd., Malvern, UK) (Rabia et al., 2020). Formulae were diluted utilizing distilled water before the measurements to ensure that the light scattering intensity was within the instrument's sensitivity range (Albash et al., 2019). ZP measurement was investigated by exploring the electrophoretic mobility of the particles in the electric field. Each sample was assessed three times, with the average value being recorded (Albash et al., 2020).

#### Experimental design and selection of the optimum TNs

The D-optimal design was utilized to assess the impact of different independent factors in fabricating TNs. Three variables were evaluated: Span 60 amount (*X*_1_), oleic acid amount (*X*_2_), and Brij^®^ type (*X*_3_). As dependent variables, EE%, PS, PDI, and ZP were chosen (Table 1). Each parameter was subjected to an analysis of variance (ANOVA) test applying Design-Expert^®^ software (Version 7, Stat-Ease Inc., Minneapolis, MN). Statistically significant *p* values <.05 were used. The system was optimized to recommend the formulation with the lowest (PS and PDI) and the greatest EE% and ZP values. The recommendation was based on the desirability function which allows all constraints to be examined at the same time. The formulation with the highest desirability outcome was chosen. The recommended optimum formula was fabricated, tested, and compared to the predicted data to ensure that the model performance is accurate.

**Table 1.** D-optimal design used for optimization of TNs formulae.

Factors (independent variables)	Levels	
	Low	High
*X*_1_: Span 60 amount (mg)	50	90
*X*_2_: Oleic acid amount (mg)	10	50
*X*_3_: Brij^®^ type	Brij 93	Brij 58

Responses (dependent variables)	Desirability constraints
*Y*_1_: EE%	Maximize
*Y*_2_: PS (nm)	Minimize
*Y*_3_: PDI	Minimize
*Y*_4_: ZP (mV)	Maximize (as absolute value)

TNs: trans-novasomes; EE%: entrapment efficiency percent; PS: particle size; PDI: polydispersity index; ZP: zeta potential.

### *In silico* studies

#### Docking studies

All the docking assessments were evaluated applying the molecular operating environment (MOE) software version 2019.0102. All compounds were sketched using ChemDraw 20.1.1 and then converted to 3D structures by MOE (Vilar et al., 2008). Prior to commencing the docking, the energy of the five compounds (FTN, cholesterol, Span 60, oleic acid, and Brij 93) was minimized using AMBER10:EHT. Each of the formula components (cholesterol, Span 60, oleic acid, and Brij 93) was solvated and used as a potential receptor to bind with FTN. Finally, the docking studies were conducted at the maximum accuracy using induced fit protocol and triangle matcher as a scoring function. The docking results were analyzed based on both docking scores and molecular interactions.

#### Molecular dynamics simulation (MDS)

All MDS was done applying MOE and NAMD 2.1.1 Software (Phillips et al., 2005). Three MDS experiments were carried out to verify the docking evaluation results. The three experiments were performed on FTN, cholesterol, and FTN–cholesterol complex. The conditions for NAMD 2.1.1 were prepared using MOE which included solvation, Langevin dynamics, temperature, pressure, and periodic boundary conditions. The three systems were energy minimized applying the steepest descent minimization algorithm under AMBER10:EHT until 0.1 RMS kcal/mole/A2 is reached (Case et al., 2005; El-Hassab et al., 2018, 2020). For 5 ns, the energy-minimized structures were equilibrated. To obtain the long-range electrostatic value, the particle mesh Ewald (PME) method with a 12 Å cutoff was implemented. The three equilibrated systems entered the production phase with no restraints for 100 ns and a time step of 2 fs, with structural coordinates saved every 50 ps. The root means square deviation (RMSD) of all heavy atoms in the system was calculated using the observed trajectories from the production phase.

### Transmission electron microscopy (TEM)

TEM (Joel JEM 1230, Tokyo, Japan) was utilized to detect the optimum TNs morphologically. A negatively stained sample with an aqueous solution of phosphotungstic acid (Khan et al., 2022) was applied to a copper-covered carbon grid and let for drying until TEM examinations were performed (Basha et al., 2013).

### *In vitro* release of the optimum TNs

*In vitro* drug release was evaluated by United States Pharmacopeia (USP) dissolution apparatus II (Pharma Test, Hainburg, Germany) at 37 °C for 24 hours. One milliliter from the optimum TNs were placed in tubes with a permeation area of 3.14 cm^2^ with one end tightly covered with a cellulose membrane and the other end attached to the shaft of the dissolution apparatus instead of the baskets. Fifty milliliters phosphate buffer saline solution was used as the receptor medium (pH 5.5). At 1, 2, 3, 4, 5, 6, 8, and 24 hours, aliquots were removed. A UV spectrophotometer was utilized to examine the samples at *λ*_max_ 252 nm (Albash et al., 2021). All of the measurements were done in triplicate ± SD.

### The impact of storage on the optimum TNs

The optimum TNs were maintained at 4–8 °C for 45 days to examine its physical stability (Abd-Elsalam et al., 2018). Before and after storage, the optimum TNs were examined for changes in appearance, EE%, PS, PDI, and ZP. The obtained data were statistically inspected applying Student’s *t*-test via SPSS^®^ program 22.0 (SPSS Inc., Chicago, IL).

### Assessment of minimum inhibitory concentration (MIC)

The technique was conducted using XTT reduction method. The MIC value is the minimum concentrations at which *Trichophyton mentagrophytes* (ATCC 9533) growth is fully inhibited. A micro-well dilution technique was employed to estimate MIC applying generated inoculums containing 10^4^ CFU/mL. In a 96-well plate, DMSO was utilized to dilute FTN suspension and optimum TNs. Forty microliters of sabouraud (the growth medium), 10 µL inoculum, and 50 µL of diluted formulae were placed in every well of the microplate. As a negative control, DMSO was employed. The plates were incubated for 24 h at 37 °C before being exposed to 40 µL of XTT and after that incubated in the dark at the same temperature for an hour. The microplate photometer (Tecan Sunrise absorbance reader, Reading, UK) was employed to calculate any color change caused by XTT decrease at 492 nm. The following is the equation used to determine inhibition % (Abdelbari et al., 2021):

(2) $$\text{Inhibition \% }=1-(\frac{\text{mean of test wells}}{\text{mean of control wells}})\times \;100$$

### Clinical appraisal

The clinical appraisal was carried out in compliance with the ethical requirements of the Declaration of Helsinki. The Institutional Review Board, Faculty of Medicine, Minia University has reviewed and approved the research ethics committee for clinical studies (approval no. 41:5/2021).

#### Patients

This clinical appraisal included 40 patients having lesions of tinea corporis. Those patients were picked from the Minia University Hospital (Dermatology Clinic). Before participating in this ongoing study, each patient signed an informed written consent form. Only patients with skin lesions who had not received any topical or systemic treatments for at least one month prior to participation were included. However, patients with skin lesions, showing tinea capitis or onychomycosis, patients with uncontrolled diabetes, pregnant or lactating females; were excluded.

All of the patients provided a complete personal history (age, gender, and occupation), current history (onset, course, and duration), previous history (previous treatment), and family history of fungal infections. The patients were photographed before treatment and after 4 weeks or complete improvement. The lesions were subjected to dermoscopic examination before treatment and after 4 weeks or complete cure. Skin scrapings were collected from the lesions for mycological diagnosis before treatment and after 4 weeks or complete improvement. Two drops of 20% potassium hydroxide (KOH) were added to the collected skin scrapings on a glass slide prior to its microscopic examination (Sobera & Elewski, 2008).

Patients were allocated into two equal groups, 20 patients each. In group A, patients were instructed to apply the optimum TNs formulation (25 mg FTN) twice daily for 4 weeks or complete cure. In group B, each patient was instructed to apply Miconaz cream^®^ (miconazole nitrate 2%) in the same manner as group A (twice a day for 4 weeks or complete cure). The simultaneous use of any topical, systemic antifungal, systemic antihistamine, or systemic corticosteroid agent other than the received treatment was not permitted. Throughout the study, patients were instructed to record any discomfort. The efficacy of the used treatment was measured using three parameters: clinical cure, dermoscopic cure, and mycological cure (McNeely & Spencer, 1998; Jerajani et al., 2000).

#### Clinical cure

Erythema, pruritus, and scaling are the measured parameters in each lesion. They were graded into (1) mild, (2) moderate, and (3) severe and were scored at baseline (day 0) and after 4 weeks. The maximum score was 9. Moreover, the global evaluation response (Jerajani et al., 2000; Verma and Hefferenan 2003) was evaluated per visit and is distinguished as (1) cleared: complete remission of clinical signs and symptoms except for residual manifestations; (2) excellent: clinical signs and symptoms of baseline improved by 90–99%; (3) good: clinical signs and symptoms of baseline improved by 50–89%; (4) fair: clinical signs and symptoms of baseline improved by 25–49%; (5) poor: clinical signs and symptoms of baseline improved by <25%; (6) worse: clinical signs have deteriorated since the beginning of the study.

#### Dermoscopic cure

The dermoscopic examination was done for all the patients before and 4 weeks after treatment or complete cure, the efficacy of the assessment was scaled on a two-point scale, with 0 indicating the absence of dermoscopic criteria and 1 indicating the existence of dermoscopic criteria. The dermoscopic criteria of tinea corporis mentioned in the literature included follicular micropustules, diffuse erythema, and brown spots surrounded by a white-yellowish halo, wavy hair, broken hair, and, in rare cases, morse code hair (Gupta et al., 2003).

#### Mycological cure

The direct mycological examination was repeated after 4 weeks or after complete cure in both groups (A and B) through a light microscope (Accu-Scope, Olympus, Tokyo, Japan) with a camera (E-330 SLR, Olympus, Tokyo, Japan). The mycological cure indicates the disappearance of all fungal elements from the skin scrapings. The assessment efficacy was scaled on a two-point scale, with 0 representing the absence of fungal elements and 1 representing the existence of fungal elements.

#### Statistical analysis

Mann–Whitney’s test, Wilcoxon’s signed rank test, and Chi-Square test were utilized for clinical experiments via SPSS^®^ program 22.0. At *p*≤ .05, the difference is significant.

## Results and discussion

### Analysis of D-optimal design

**Figure 1.**
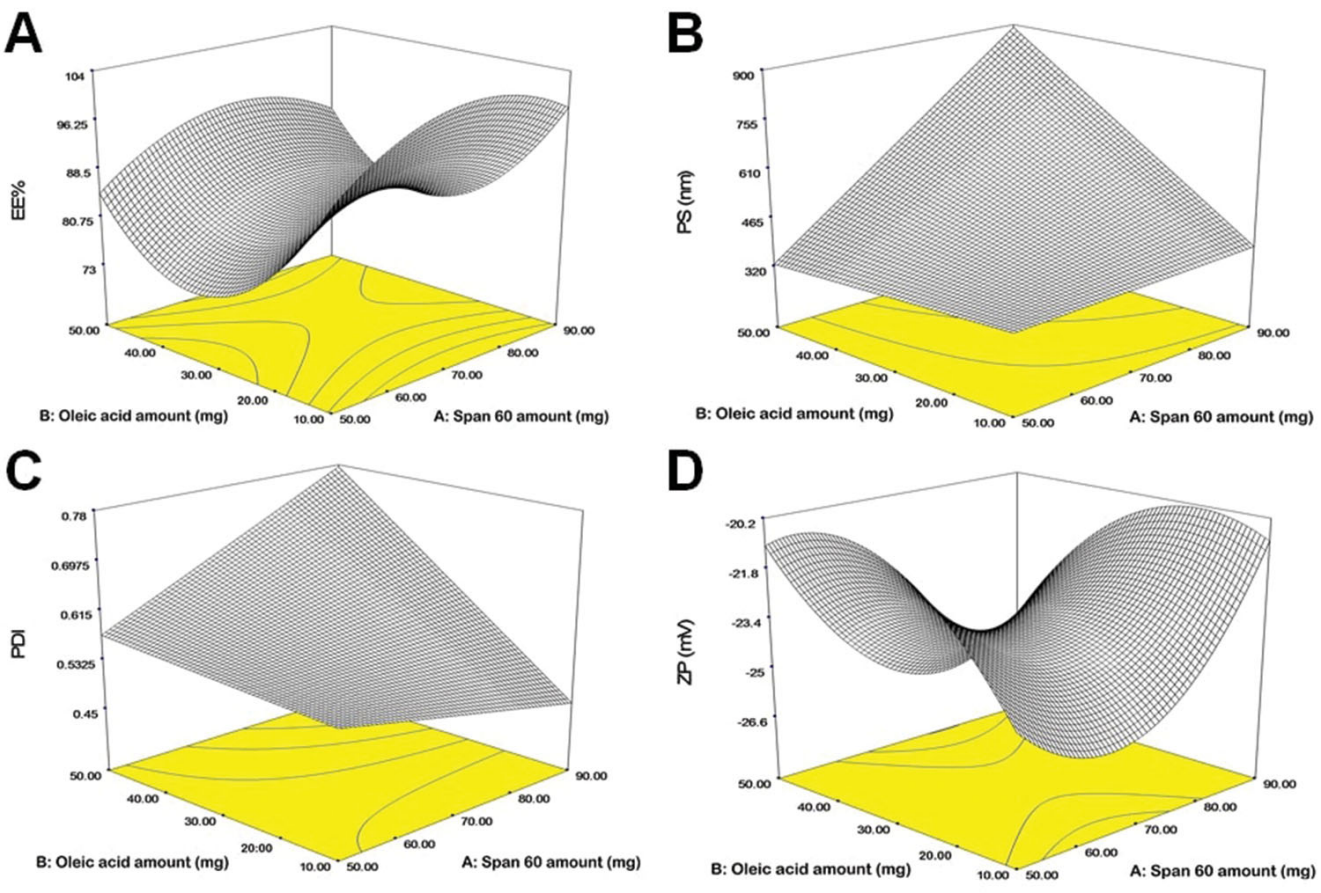
The impact of formulation variables on (A) EE%, (B) PS, (C) PDI, and (D) ZP of FTN loaded TNs. EE%: entrapment efficiency percent; PS: particle size; PDI: polydispersity index; ZP: zeta potential; FTN: fenticonazole nitrate; TNs: trans-novasomes.

Screening experiments were implemented to assess the independent variables that might have an impact on the development of FTN enclosed TNs. The preparation of TNs was done by the use of the Design Expert^®^ program according to D-optimal design. The created design recommended 15 experimental runs to reflect all conceivable combinations of the various levels of the examined factors. The chosen model was a quadratic model for EE% and ZP and was 2FI for PS and PDI. The potential of the model to navigate the design space was confirmed with adequate precision. A ratio of more than four is preferable (Albash et al., 2019), as evidenced by all the explored responses (Table 2). The predicted (*R*^2^) values matched adjusted (*R*^2^) values in all of the investigated responses. The impact of formulae factors on the explored responses is displayed as 3D plots in Figure 1(A–D).

**Table 2.** D-optimal analysis outcome of TNs formulae and the predicted and observed responses of the optimum TNs (F7).

Responses	*R* ^2^	Adjusted *R*^2^	Predicted *R*^2^	Adequate precision	Significant factors
EE%	0.98	0.95	0.78	16.69	*X*_1_, *X*_2_, *X*_3_
PS (nm)	0.97	0.96	0.90	20.30	*X*_1_, *X*_2_, *X*_3_
PDI	0.73	0.52	−0.74	6.26	*X*_2_
ZP (mV)	0.98	0.96	0.77	22.87	*X*_3_

Response	*Y*_1_ EE%	*Y*_2_ PS (nm)	*Y*_3_ PDI	*Y*_4_ ZP (mV)
Observed values	100.00%	358.60	0.51	–30.00
Predicted values	100.00%	431.18	0.57	–30.14

TNs: trans-novasomes; EE%: entrapment efficiency percent; PS: particle size; PDI: polydispersity index; ZP: zeta potential.

#### The impact of formulation variables on the EE%

A critical restriction in the treatment of fungal infections on the skin is the ability of TNs to entrap a large percentage of FTN. The percentage of FTN enclosed within TNs fluctuated between 81.00 ± 1.25 and 100.00 ± 1.10% (Table 3). In the present work (Figure 1(A)), it was noticed that Span 60 amount (*X*_1_) significantly (*p*=.0013) impacted EE% of FTN. The obtained findings indicated that as Span 60 amount was increased, the EE% value augmented. This might be ascribed to the increase in emulsification ability caused by the high Span 60 amount in TNs. Similar outcomes were observed by Mosallam et al. (2021a) in a study on the fabrication of novasomes for the topical delivery of terconazole. In addition, Span 60 with high transition temperature and HLB of 4.7 promoted high capability to reserve a more organized gel structure and well-defined TNs that prohibited FTN leakage to the aqueous phase (Vora et al., 1998).

**Table 3.** Experimental runs, independent variables, and measured responses of D-optimal design of TNs formulae.

Formulations	*X*_1_ Span 60 amount (mg)	*X*_2_ Oleic acid amount (mg)	*X*_3_ Brij^®^ type	*Y*_1_ EE%	*Y*_2_ PS (nm)	*Y*_3_ PDI	*Y*_4_ ZP (mV)
F1	50	50	Brij 93	86.94 ± 4.14	288.10 ± 9.78	0.53 ± 0.08	–22.90 ± 0.36
F2	50	50	Brij 93	86.00 ± 4.20	288.10 ± 10.01	0.53 ± 0.07	–22.00 ± 0.21
F3	50	10	Brij 93	90.48 ± 2.37	430.70 ± 47.38	0.54 ± 0.08	–29.00 ± 0.72
F4	50	10	Brij 58	96.74 ± 1.39	331.80 ± 16.33	0.54 ± 0.07	–20.50 ± 0.17
F5	50	10	Brij 58	96.00 ± 1.56	331.80 ± 20.65	0.53 ± 0.07	–20.00 ± 0.20
F6	50	50	Brij 58	82.65 ± 1.31	376.50 ± 43.33	0.64 ± 0.02	–19.50 ± 0.70
F7	70	10	Brij 93	100.00 ± 1.10	358.60 ± 10.76	0.51 ± 0.004	–30.00 ± 0.80
F8	70	50	Brij 58	90.00 ± 3.58	560.00 ± 8.90	0.59 ± 0.01	–23.90 ± 0.26
F9	70	30	Brij 93	84.73 ± 19.02	507.50 ± 93.02	0.57 ± 0.08	–27.50 ± 1.20
F10	70	20	Brij 58	89.97 ± 1.64	455.30 ± 23.57	0.64 ± 0.05	–20.00 ± 0.80
F11	90	50	Brij 93	98.81 ± 7.75	964.80 ± 48.86	0.76 ± 0.09	–28.20 ± 0.87
F12	90	10	Brij 58	98.90 ± 0.98	235.10 ± 4.12	0.23 ± 0.002	–17.40 ± 0.56
F13	90	10	Brij 93	98.21 ± 1.04	542.70 ± 31.52	0.68 ± 0.01	–24.50 ± 1.50
F14	90	50	Brij 93	96.17 ± 3.07	964.80 ± 48.63	0.75 ± 0.09	–28.20 ± 0.87
F15	90	50	Brij 58	81.00 ± 1.25	825.70 ± 37.05	0.88 ± 0.05	–21.90 ± 0.35

FTN: fenticonazole nitrate; TNs: trans-novasomes; EE%: entrapment efficiency percent; PS: particle size; PDI: polydispersity index; ZP: zeta potential.

All the formulated vesicles included the same amounts of FTN, cholesterol, and Brij^®^. Data are displayed as mean ± SD (*n* = 3).

Regarding oleic acid amount (*X*_2_), it was found that increasing oleic acid amount resulted in a significant (*p*<.0001) decrease in the EE%. Kumar et al. (2018) reported when the oleic acid amount was increased, the EE% of minoxidil decreased. This might be due to the presence of the excessive amount of oleic acid that contributes to the destruction of the lipid matrix of TNs and hence EE% decreases (Zhang et al., 2013). ANOVA disclosed that Brij^®^ type (*X*_3_) had a significant (*p*=.0073) influence on EE%. The observed outcome revealed higher EE% values in TNs containing Brij 93 compared to Brij 58 which could be explained by the difference in HLB values for both Brij 93 (HLB = 4) and Brij 58 (HLB = 15.7) (Mosallam et al., 2021b). Albash et al. (2019) reported that an edge activator with a lower HLB value (greater hydrophobicity), when used with the hydrophobic drug, might result in a higher drug EE% compared to edge activators with lower HLB values.

#### The impact of formulation variables on the PS

PS is one of the most important factors determining drug infiltration via the skin (Verma et al., 2003). The use of nanosystem technology enables better penetration into the skin layers (Du Plessis et al., 1994). PS of the fabricated TNs fluctuated between 235.10 ± 4.12 and 964.80 ± 48.63 nm (Table 3). Figure 1(B) displays that Span 60 amount (*X*_1_) impacted the PS of the fabricated TNs significantly (*p*<.0001). The outcomes revealed that the PS of the fabricated TNs increased upon increasing Span 60 amount. This corresponded to the EE% outcomes as Span 60 showed high EE% values at high concentrations. Hathout et al. (2007) observed that the higher drug enclosed within the vesicles could cause gap formation through the bilayers to widen resulting in an increase in PS. Additionally, the alkyl chain length of Span 60 is 18 carbon atoms that might result in a larger core space of the TNs with a greater diameter (Al-Mahallawi et al., 2014).

Considering oleic acid amount (*X*_2_), a significant (*p*<.0001) increase in the PS was observed with increasing oleic acid amount. Due to the attractive steric forces, an increase in the chain length of oleic acid reduces the ability of oil molecules to penetrate the interfacial film, resulting in larger particles (Sarheed et al., 2020). This outcome came in agreement with a report by (Leung & Shah, 1987). Moreover, Shukla et al. (2003) observed that the angular structure of oleic acid might lead to larger particles. According to statistical analysis, Brij^®^ type (*X*_3_) had a significant (*p*<.0001) impact on the PS, where Brij 93 produced larger PS compared to Brij 58. This might be related to the PEG content of both edge activators as Brij 93 contained 2 PEG units and Brij 58 contained 20 PEG units (Tagami et al., 2011). It was suggested that lowering the PEG content of the PEGylated edge activator could augment the rate of vesicles’ precipitation and vesicles’ agglomeration resulting in larger PS (Caliceti et al., 2004).

#### The impact of formulation variables on the PDI

The quality of a nanosystem is determined by the PDI values of the nano-dispersion. A PDI of zero indicates uniform dispersion with a narrow PS range. A PDI of zero represents homogenous dispersion with a small PS range. On the contrary, a value near 1 resembles a polydisperse population (Stetefeld et al., 2016). All of the developed TNs had PDI values ranging from 0.23 ± 0.002 to 0.88 ± 0.05 (Table 3) indicating that some of the prepared TNs were polydisperse (Figure 1(C)). However, only TNs with a narrow range of PS were considered for optimization. ANOVA displayed that only oleic acid amount (*X*_2_) had a significant (*p*=.017) impact on the PDI. On the other hand, Span 60 amount (*X*_1_) and Brij^®^ type (*X*_3_) had no significant effect on the PDI (*p*=.402 and .616, respectively). Regarding oleic acid amount (*X*_2_), it was clear that as the amount of oleic acid was increased, the PDI increased. This might be justified by there was a direct correlation found between the PS and the PDI, with the highest PDI value found in TNs with the highest PS. This outcome was in accordance with the previous finding of Kumar et al. (2018) working on minoxidil loaded oleic acid vesicles.

#### The impact of formulation variables on the ZP

The optimal charged surface can promote physical stability via establishing electrostatic repulsion between the vesicles, which protects them from accumulation or fusion (Ruckmani & Sankar, 2010). The fabricated TNs demonstrated a negative charge ZP (Figure 1(D)) value varied from −17.40 ± 0.56 to −30.00 ± 0.80 mV (Table 3). It was reported by Mosallam et al. (2021a) that novasomes possess a high negative charge surface related to the existence of free fatty acids on vesicles surface. Oleic acid molecules contain an ionizable carboxylic group. Thereby, it is considered an ionic lipid on its own. Similar outcomes were observed by Manconi et al. (2011) who found that the highest negative charge in vesicles was observed when oleic acid was used during vesicles formation.

Statistical analysis displayed that only Brij^®^ type (*X*_3_) had a significant (*p*<.0001) influence on the ZP. On the contrary, Span 60 amount (*X*_1_) and oleic acid amount (*X*_2_) had no significant impact on the ZP (*p*=.6257 and .3918, respectively). With respect to Brij^®^ type (*X*_3_), when comparing Brij 93 with Brij 58, it was found that Brij 93 had a greater negative ZP value. This might be ascribed to the fact that Brij 58 (HLB = 15.7) is more hydrophilic than Brij 93 (HLB = 4) which could result in the negative charge being shielded by adhering to the surface of the bilayers of TNs, disguising its charge. As a result, the ZP value has been significantly reduced (Wilson et al., 2008; Aziz et al., 2018). Moreover, as previously mentioned Brij 93 contained 2 PEG units and Brij 58 contained 20 PEG units, respectively. The higher concentration of hydrophilic PEG steric shield slightly screened the surface charge of TNs at the vesiclesʼ surface resulting in lower ZP (Hu et al., 2007).

#### Determination of the optimum TNs

Numerical analysis using the Design-Expert^®^ program was utilized to locate the optimum TNs formula that has the following characteristics: the highest EE%, highest absolute value of ZP, lowest PS, and PDI. The program selected F7 as the optimum formulation with the greatest desirability value (0.85). The chosen formulation was fabricated employing 70 mg Span 60, 10 mg oleic acid, and 10 mg Brij 93. The predicted outcomes of F7 were consistent with the actual outcomes (Table 2). Thereby, F7 was chosen for further investigations.

### *In silico* studies

#### Docking studies

**Figure 2.**
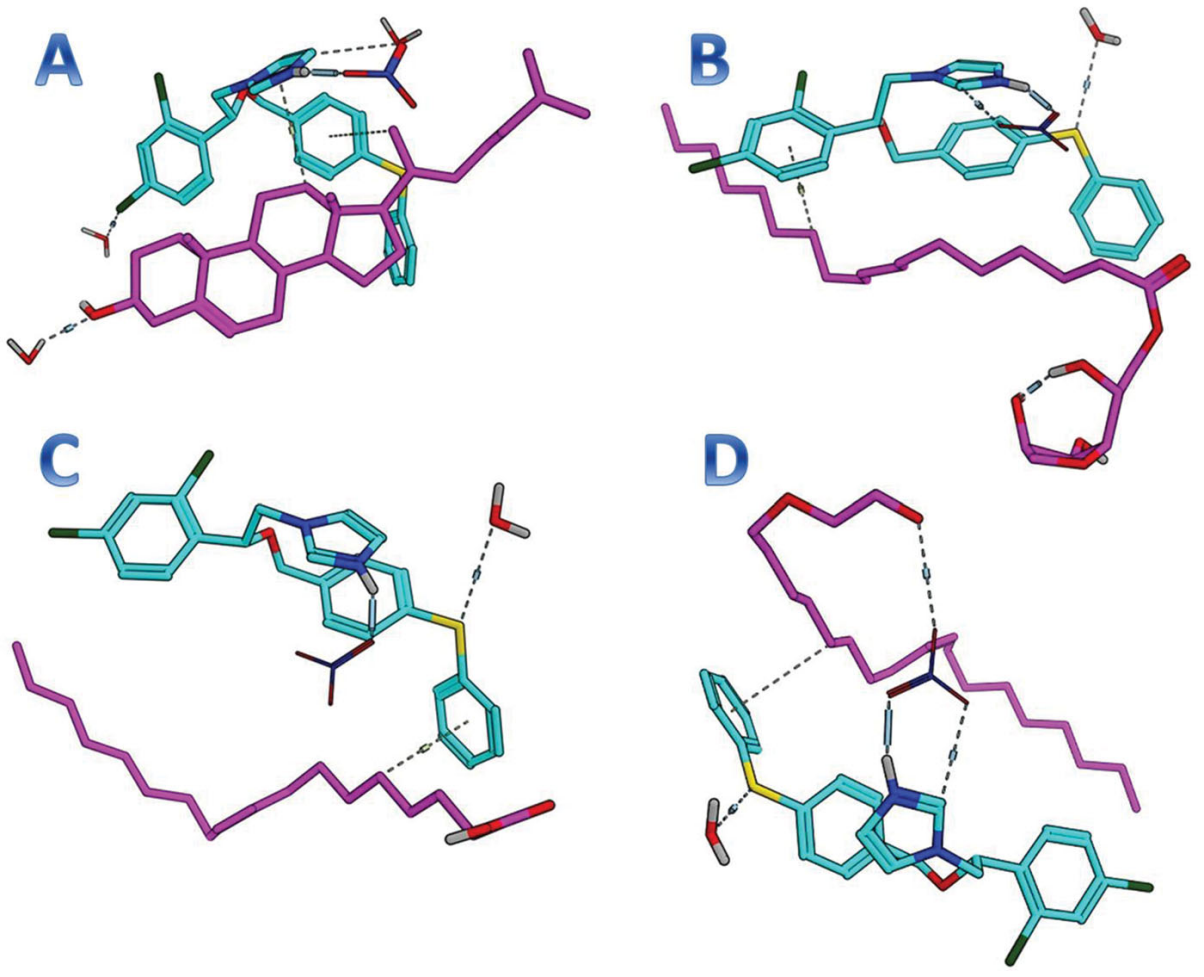
Docking results for FTN with (A) cholesterol, (B) Span 60, (C) oleic acid, and (D) Brij 93. FTN: fenticonazole nitrate.

The purpose of this section was to explore the possible interaction between the formula components and FTN. Accordingly, each of the formula components was considered as a potential receptor for FTN. As early mentioned, the water is the final solvent on the formula, therefore, each component was solvated prior to the conduction of the docking. Interestingly, FTN was able to induce favor binding with the four components of the formula. It achieved binding scores of −5.9, −4.1, −3.6, and −4.6 kcal/mole with cholesterol, Span 60, oleic acid, and Brij 93, respectively. As depicted in Figure 2, FTN was engaged in multiple interactions with the four components. The docking results highlighted the potential role of cholesterol to bind efficiently with FTN. In this context, the complex of FTN–cholesterol was subjected to molecular dynamics for further endorsement. The observed outcomes could justify the importance of the addition of cholesterol in TNs. Moreover, cholesterol would act as a carrier maintaining the loading of FTN inside TNs.

#### Molecular dynamics simulation

**Figure 3.**
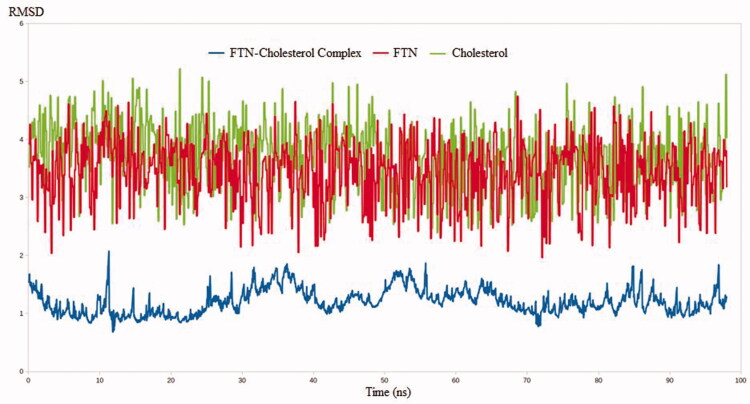
The RMSD analysis of the MDS results throughout 100 ns. FTN: fenticonazole nitrate; RMSD: root means square deviation; MDS: molecular dynamics simulation.

The MDS technique has been validated in a number of drug discovery studies. The technique has been successfully implemented in the evaluation of binding stability between a ligand and its target through the accurate determination of complex dynamicity and binding strength (El Hassab et al., 2020; Alamri et al., 2021; El Hassab et al., 2021). Furthermore, unlike docking techniques, MDS calculations are based on millions of conformations making it a superior technique over many drug design tools. In this context, three MDS experiments use FTN, cholesterol, and FTN–cholesterol complex. The RMSD values were calculated for the three systems to estimate the binding stability between FTN and cholesterol relative to the free form of each one. As expected, the RMSD values for the FTN–cholesterol complex were much less than either FTN or cholesterol alone. As shown in Figure 3, the RMSD values reached 1.7, 3.9, and 4.3 nm for FTN cholesterol complex, FTN, and cholesterol, respectively. Worth noting, conformational sampling of the FTN–cholesterol complex revealed the ability of the complex to maintain favorable interactions throughout the entire simulation.

### Transmission electron microscopy

**Figure 4.**
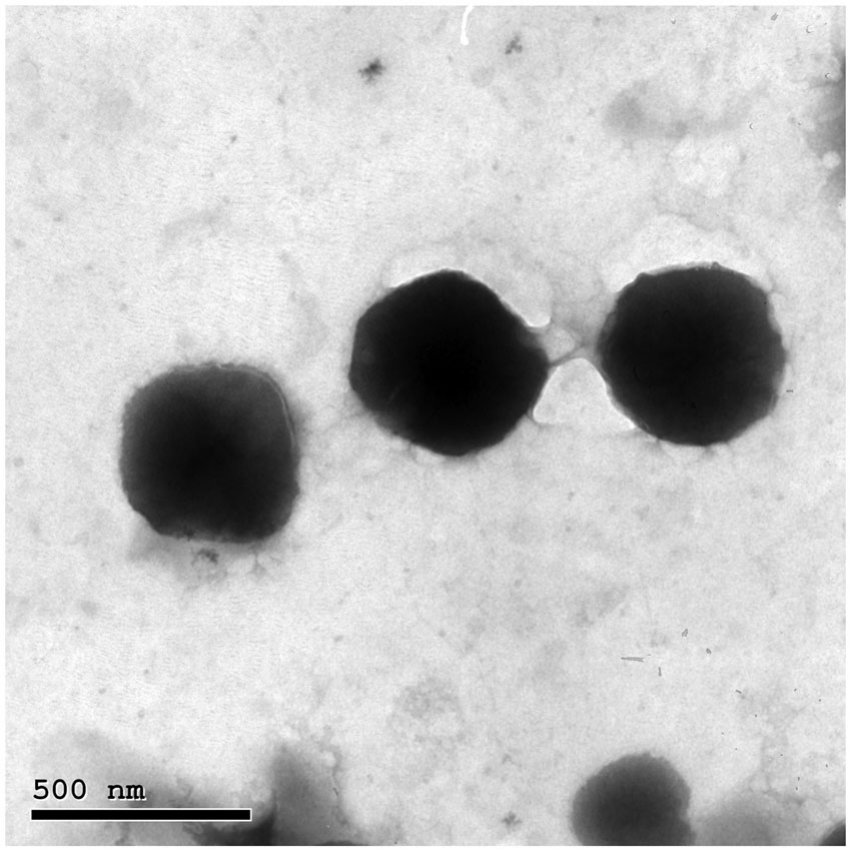
Morphology of the optimum TNs (F7). TNs: trans-novasomes.

The TEM micrograph of the optimum TNs (F7) displayed a spherical shape with the development of smooth, non-aggregated, and small vesicles (Figure 4). The PS of F7 assessed by zetasizer agreed well with TEM measurements.

### *In vitro* release of the optimum TNs

The *in vitro* release profile of FTN from the optimum TNs (F7) in comparison with FTN suspension is illustrated in the supplementary materials (Figure 1). The release of FTN from F7 is obviously greater than that from the FTN suspension. This might be related to the ability of Brij^®^ to solubilize FTN due to its surface-active aspects (Albash et al., 2019), resulting in increased solubility of the entrapped sparsely water-soluble antifungal drug, FTN.

### The impact of storage on the optimum TNs

After 45 days of short-term stability investigation, F7 showed no difference in appearance, EE%, PS, PDI, and ZP. These outcomes demonstrated that the manufactured TNs were physically stable which might be owing to the steric stabilization provided by Brij 93 due to the presence of PEG that stabilized TNs by steric hindrance (Abdelbary et al., 2019). In addition, the highly negatively charged TNs acquired by the presence of oleic acid increased the ZP of TNs and prohibited their coalescence.

### Assessment of minimum inhibitory concentration

**Figure 5.**
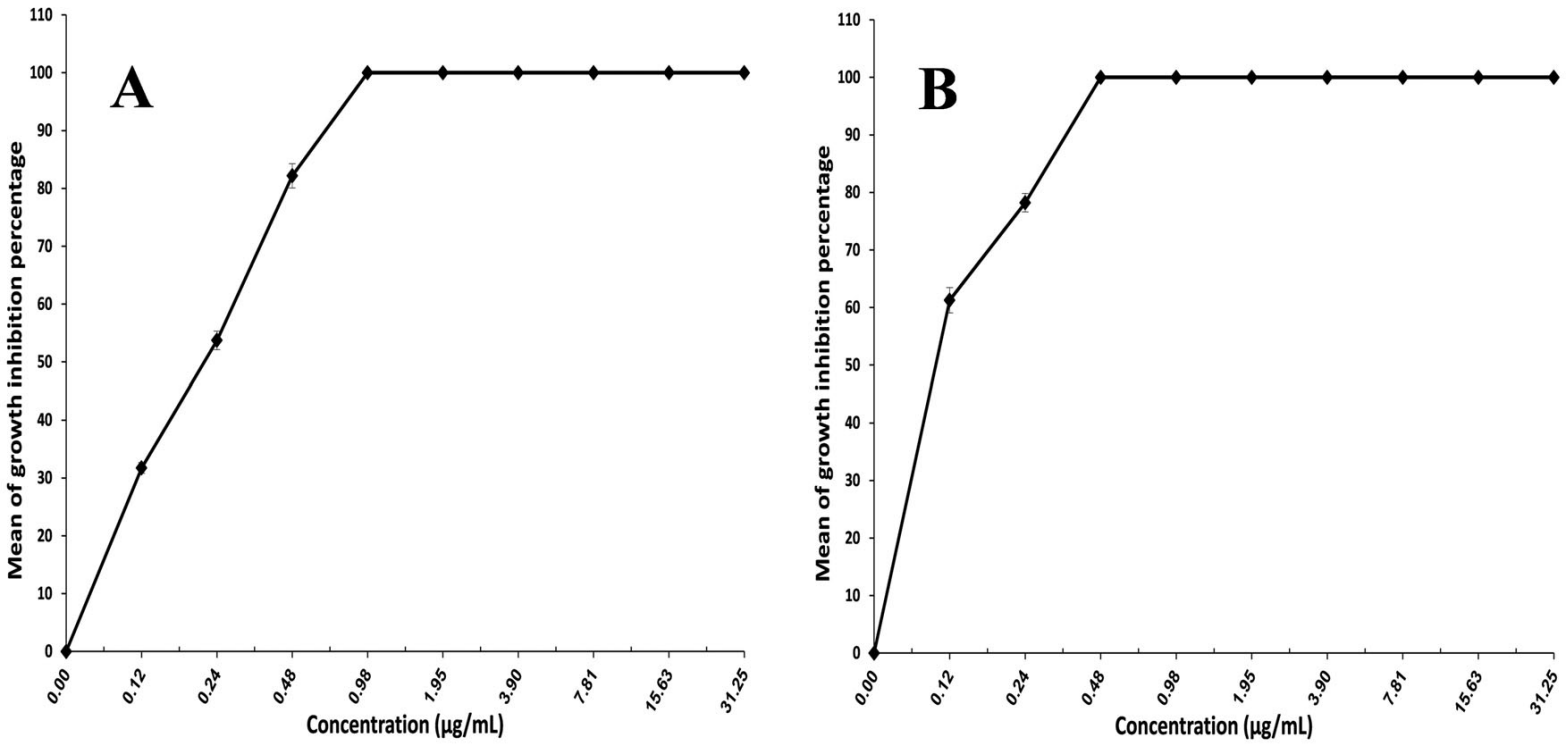
Microbiological efficacy of (A) FTN suspension and (B) F7 for the treatment of *Trichophyton mentagrophytes* infection. Data are presented as mean ± SD (*n* = 3). FTN: fenticonazole nitrate.

*Trichophyton mentagrophytes* was chosen for this test, as it is considered the most common causal agent for dermatophytosis (Frías-De-León et al., 2020; Petrucelli et al., 2020). The ability of XTT reduction assay to quantify the activity of *Trichophyton mentagrophytes* provides an advantage over the agar diffusion technique. It assesses cell activity using a quantitative colorimetric assessment of the intracellular formazan molecule produced when XTT is reduced (Roehm et al., 1991; Jahn et al., 1995). Figure 5 depicts the antifungal activity of FTN suspension and F7. The MIC for F7 (0.48 µg/mL) was lower than that of FTN suspension (0.98 µg/mL). The formulation's effectiveness increases as the MIC value decreases. F7 achieved a twofold reduction in the MIC when compared to FTN suspension which might be explained by high discharge and ultimate diffusion of FTN from F7, as well as high oxidative stress caused by the inclusion of polyunsaturated lipids in the membrane will contribute to the antifungal activity of oleic acid when compared to FTN suspension (Avis & Bélanger, 2001; Thibane et al., 2010; Shahin et al., 2011).

### Clinical appraisal

Demographic data of the 40 patients included in the study are described in the supplementary materials (Table 1), without a significant difference was found between the two groups. The patients were presented with tinea corporis lesions which appear as circinate erythematous scaly lesions with elevated raised border on the face, trunk, or extremities (Bhat et al., 2019). Positive KOH scraping revealed fungal elements in the form of hyphae which confirmed the diagnosis. In terms of the baseline sign and symptom score, the two groups were matched. The median sign and symptom score of tinea corporis at the baseline was 9, which was reduced to 0 by group A (received F7) while it was 9 at the baseline that became 6 at the end of the treatment in group B (received Miconaz^®^ cream) with a statistically significant difference (*p*<.001) between the two groups (Table 4(A)).

**Table 4.** Clinical score, dermoscopic cure, and mycological cure of tinea corporis in patients before and after treatment.

	Group A (received F7)	Group B (received Miconaz^®^ cream)	*p* Value
(A)			
*Clinical score*			
Before			
Median IQR	9 (8–9)	9 (8.3–9)	.807
After			
Median IQR	0 (0–0)	6 (5–6)	<.001*
*p* Value	<.001*	<.001*	
(B)			
*Dermoscopic cure*			
Before			
Score 0	0 (0%)	0 (0%)	1
Score 1	20 (100%)	20 (100%)	
After			
Score 0	20 (100%)	0 (0%)	<.001*
Score 1	0 (0%)	20 (100%)	
*p* Value	<.001*	1	
(C)			
*Mycological cure*			
Before			
–Ve	0 (0%)	0 (0%)	1
+Ve	20 (100%)	20 (100%)	
After			
–Ve	20 (100%)	0 (0%)	<.001*
+Ve	0 (0%)	20 (100%)	
*p* Value	<.001*	1	

**Figure 6.**
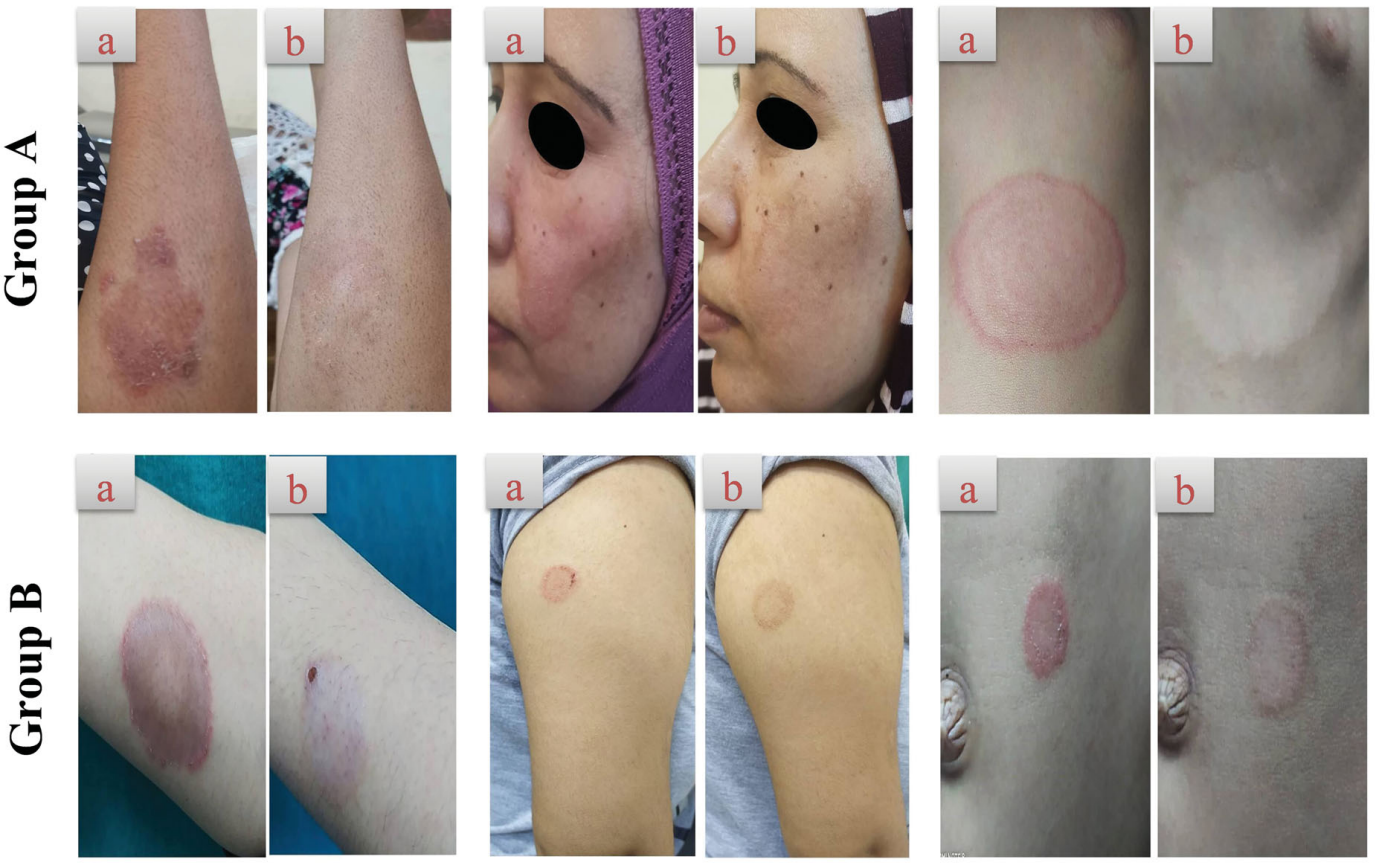
Representative patients of tinea corporis receiving F7 (group A) and Miconaz^®^ cream (group B), showing clinical evidence of efficacy of group A. (a, b) Patients before and after treatment, respectively.

**Figure 7.**
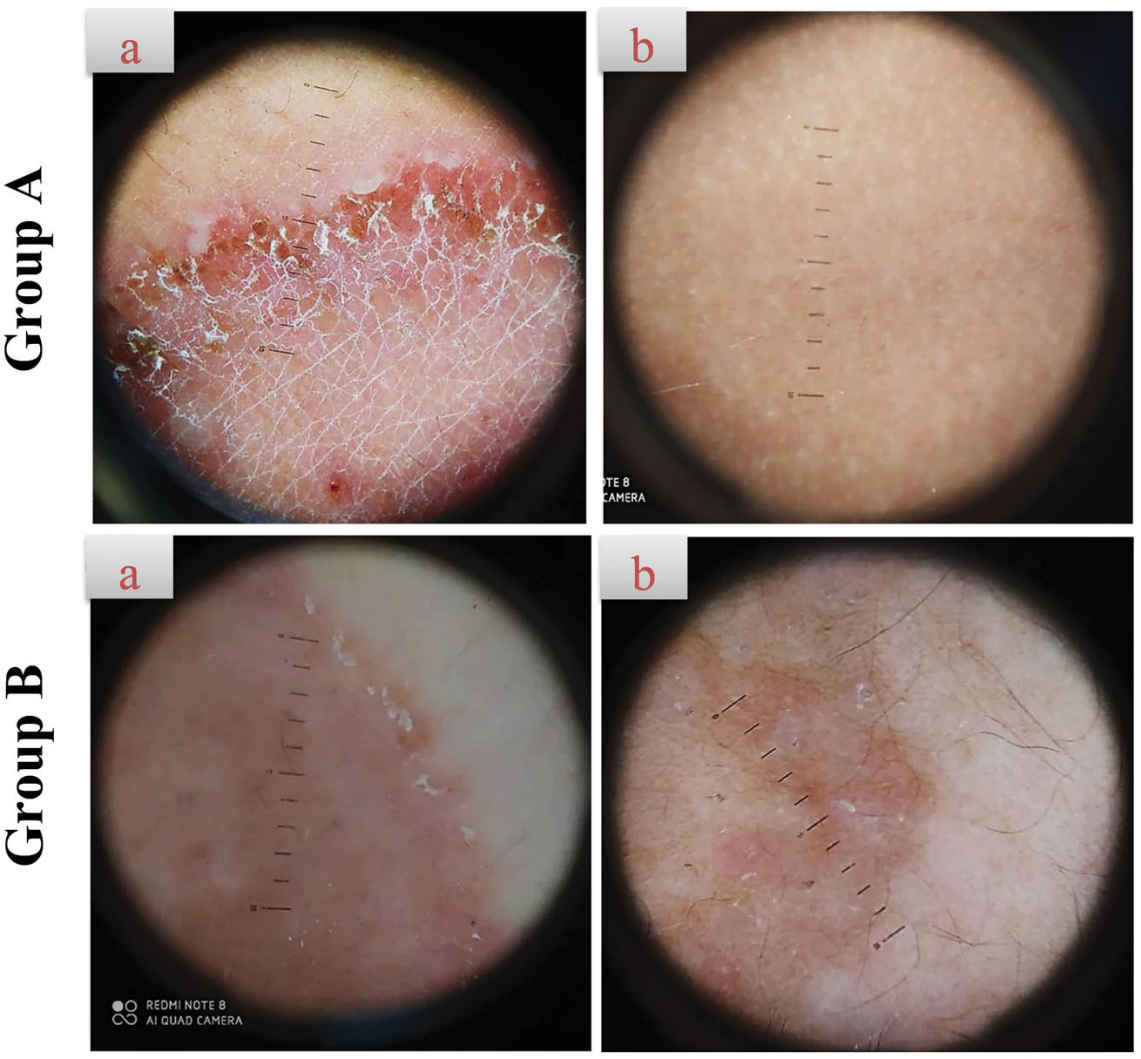
Representative dermoscopic examination of patients receiving F7 (group A) and Miconaz^®^ cream (group B), showing absence of fungal infection in group A. (a, b) Patients before and after treatment, respectively.

There was a significant clinical improvement in group A (*p*<.001) when the global evaluation response of the two groups was compared (Table 5, Figure 6). Dermoscopic evaluation became free from fungal infection after 4 weeks of therapy in group A in 100% of patients (no = 20). While in group B, the dermoscopic evaluation remained positive for the fungal criteria after 4 weeks of therapy in 100% of patients (no = 20) (Table 4(B), Figure 7). After 4 weeks of therapy in group A, 100% of patients (no = 20) had negative skin scrapings for fungal elements. While in group B, the mycological evaluation remained positive for the fungal elements after 4 weeks of therapy in 100% of patients (no = 20) (Table 4(C), Figure 6). These results indicate the superiority of the optimum TNs (F7) to Miconaz^®^ cream in treating tinea corporis. The fact that F7 was superior to Miconaz^®^ cream regarding clinical, dermoscopic, and mycological evaluation despite the former being eight times less in FTN dose (0.25% FTN) than Miconaz^®^ cream (2% miconazole nitrate) indicates the capability of TNs to improve the antifungal potential of FTN.

**Table 5.** Global evaluation response of group A (received F7) and group B (received Miconaz^®^ cream).

Global evaluation response	Group A (received F7)	Group B (received Miconaz cream)	*p* Value
Cleared	18 (90%)	0 (0%)	
Excellent	2 (10%)	0 (0%)	
Good	0 (0%)	12 (60%)	<.001*
Fair	0 (0%)	8 (40%)	
Poor	0 (0%)	0 (0%)	
Worse	0 (0%)	0 (0%)	

## Conclusions

FTN loaded TNs were fabricated utilizing the ethanol injection method, applying D-optimal design to select the optimum TNs (F7). Furthermore, F7 exhibited favorable characters in terms of EE%, size, charge, vesicular appearance, and stability. After that, the *in silico* study revealed the ability of the FTN–cholesterol complex to maintain favorable interactions throughout the entire simulation. F7 also displayed high antifungal potential against *Trichophyton mentagrophytes* relative to FTN suspension. Most importantly, F7 proved its excellence in the magnitude of clinical cure of tinea corporis when compared with Miconaz^®^ cream. Thereby, it could be verified that TNs are promising vesicles for enhancing the antifungal potential of FTN.

## Supplementary Material

Supplemental Material
