# Supplementary material for: Fenticonazole nitrate loaded trans-novasomes for effective management of tinea corporis: design characterization, in silico study, and exploratory clinical appraisal
Source: Drug Deliv. 2022 Apr 4;29(1):1100–11. doi: 10.1080/10717544.2022.2057619 (PMC8986243; doi:10.1080/10717544.2022.2057619)
Supplement: Supplemental Material [file IDRD_A_2057619_SM0260.zip › Supplementary Figures.docx]

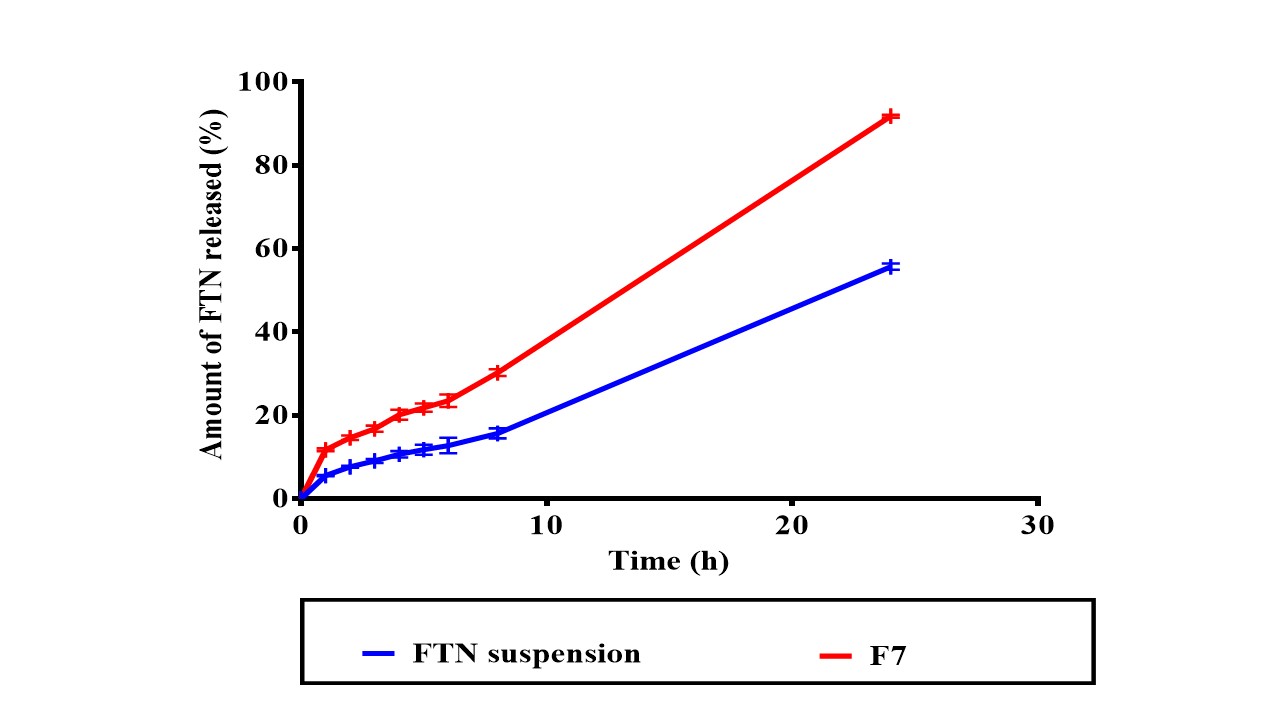
 **Supplementary Figure 1**: *In-vitro* drug release study from FTN suspension and the optimized formula.

**Abbreviation:**  FTN; Fenticonazole nitrate


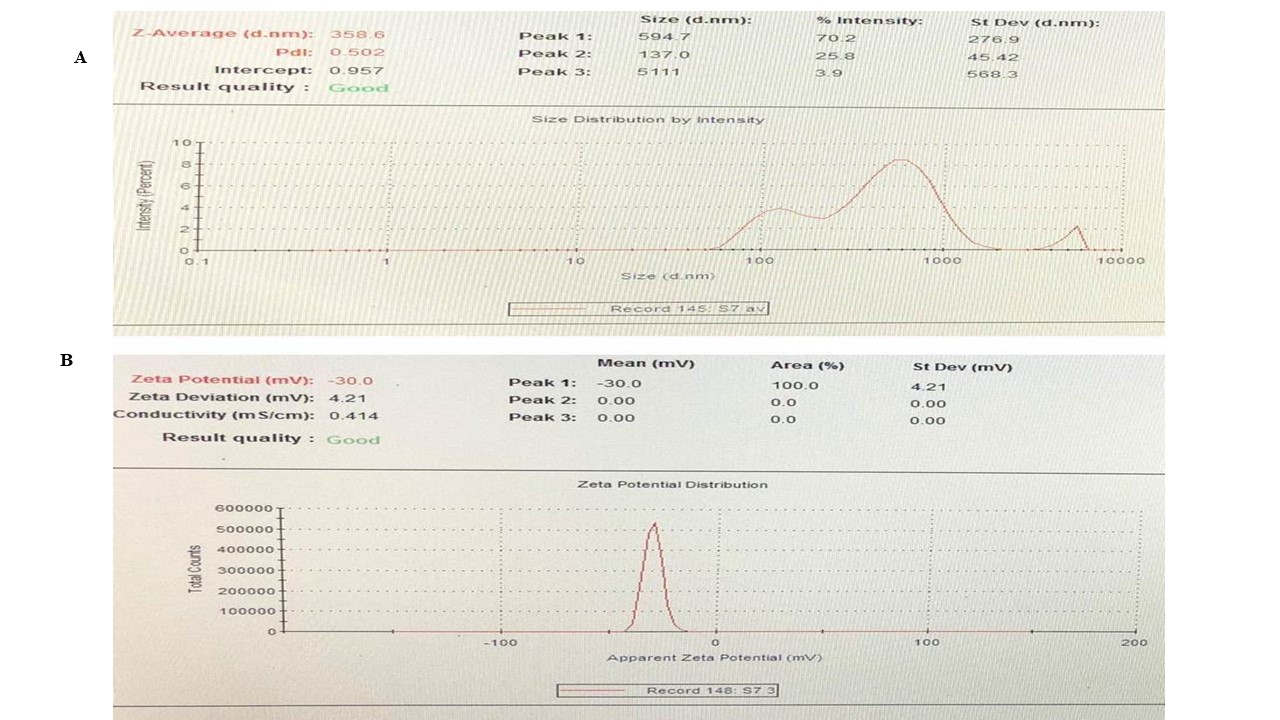


**Supplementary Figure 2:** Measurement of (a) particle size and (b) zeta potential of the optimized TNs.

Abbreviation: TNs, transnovasomes.


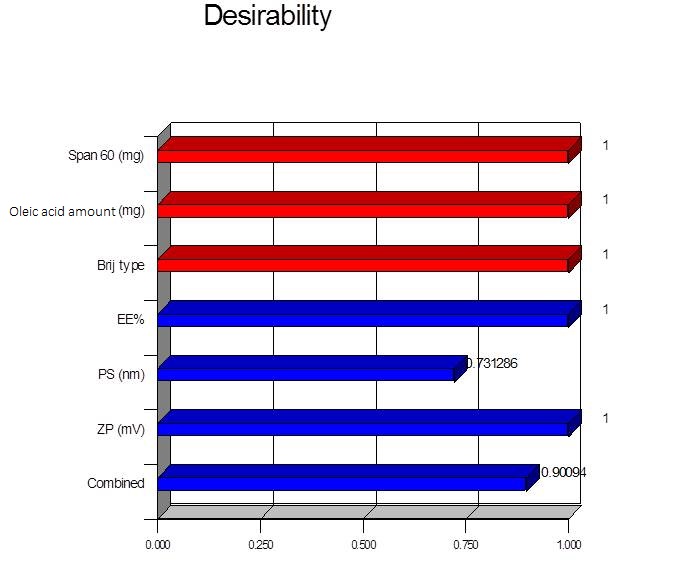


**Supplementary Figure 3:** Desirability bar plot showing desirability values of design factors, individual responses and combined responses for FTN- loaded TNs.

**Abbreviation:** FTN: Fenticonazole nitrate and TN: transnovasomes.


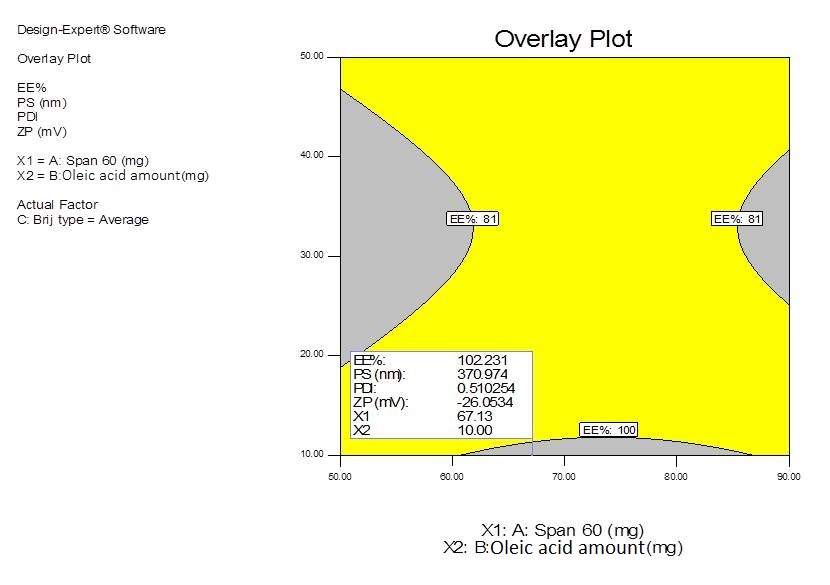


**Supplementary Figure 4**: Overlay plot for the optimization of FTN loaded TNs.

**Abbreviation:** FTN: Fenticonazole nitrate and TN: transnovasomes.
