# Supplementary material for: Fenticonazole nitrate loaded trans-novasomes for effective management of tinea corporis: design characterization, in silico study, and exploratory clinical appraisal
Source: Drug Deliv. 2022 Apr 4;29(1):1100–11. doi: 10.1080/10717544.2022.2057619 (PMC8986243; doi:10.1080/10717544.2022.2057619)
Supplement: Supplemental Material [file IDRD_A_2057619_SM0260.zip › Supplementary Table 1.docx]

**Supplementary Table 1-** Demographic data of patients received F7 (group A) and Miconaz^®^ cream (group B)

| **Group A**  **(Received F7)** | | | | **Group B**  **(Received Miconaz^®^ cream)** | | | |
| --- | --- | --- | --- | --- | --- | --- | --- |
| **No** | **Age** | **Sex** | **Duration of disease (days)** | **No** | **Age** | **Sex** | **Duration of disease (days)** |
| 1 | 10 | Male | 15 | 1 | 14 | Male | 7 |
| 2 | 12 | Male | 10 | 2 | 8 | Female | 10 |
| 3 | 9 | Female | 7 | 3 | 15 | Female | 3 |
| 4 | 22 | Male | 7 | 4 | 23 | Female | 21 |
| 5 | 16 | Female | 5 | 5 | 16 | Male | 6 |
| 6 | 8 | Female | 21 | 6 | 17 | Male | 13 |
| 7 | 25 | Male | 10 | 7 | 20 | Male | 18 |
| 8 | 11 | Male | 7 | 8 | 26 | Female | 10 |
| 9 | 15 | Female | 7 | 9 | 10 | Female | 7 |
| 10 | 6 | Male | 15 | 10 | 8 | Female | 12 |
| 11 | 9 | Female | 21 | 11 | 15 | Male | 13 |
| 12 | 10 | Male | 7 | 12 | 18 | Male | 18 |
| 13 | 11 | Female | 7 | 13 | 25 | Female | 7 |
| 14 | 13 | Male | 3 | 14 | 21 | Female | 17 |
| 15 | 18 | Female | 5 | 15 | 12 | Male | 11 |
| 16 | 24 | Female | 15 | 16 | 10 | Male | 20 |
| 17 | 5 | Male | 10 | 17 | 30 | Female | 10 |
| 18 | 20 | Female | 30 | 18 | 10 | Male | 7 |
| 19 | 8 | Female | 7 | 19 | 8 | Female | 9 |
| 20 | 15 | Male | 7 | 20 | 9 | Female | 8 |
